# Editorial: Family and Extended Kin

**DOI:** 10.3389/fsoc.2022.906081

**Published:** 2022-05-03

**Authors:** Antti O. Tanskanen, Mirkka Danielsbacka

**Affiliations:** ^1^Department of Social Research, University of Turku, Turku, Finland; ^2^Population Research Institute, Helsinki, Finland

**Keywords:** family, extended kin, relatedness, help, conflict

Who nurtured you when you were a child? In addition to mother and father, many of us would include grandparents, older siblings, aunts and uncles, kindergarten and school teachers, or neighbors. Humans are defined as “cooperative breeders,” which means that during childhood we receive a substantial amount of care and support from relatives and people who are not directly related to us (Hrdy, 2009). This is quite exceptional because in a majority of mammals, including chimpanzees, gorillas, and orangutans—our closest relatives—mothers alone are typically responsible for childrearing (Emmott and Page, 2021).

Kin networks play an important role throughout our life. Adult children continue to receive support from their parents (Szydlik, 2016), adult siblings can form extremely close bonds (Buchanan and Rotkirch, 2021), and grandparents (Tanskanen and Danielsbacka, 2019), and aunts and uncles (Milardo, 2009) can be an important part of extended family also when children reach adulthood. Although people have a tendency to form closer bonds with genetically related people, kin networks can extend beyond genetically related individuals (Salmon and Shackelford, 2011).

The family is a key social institution. It can be a primary source of happiness and healthy living, but also a source of disagreement and conflict. The present Frontiers in Sociology Research Topic considers family relationships from different angles and how these relationships shape our everyday life. This Research Topic consists of six empirical studies.

Daly and Perry consider in-law relationships and find that in-law relationships are multidimensional by nature. Because of common descendants, in-laws also have similar fitness interest with one another, which is likely to lead to the formation of cooperative relationships between family members who are not genetically related. That said, nepotistic efforts do not necessarily overlap completely, which may lead to conflict. Preliminary evidence from Bangladesh indicates that in-law conflict may increase mortality among both mothers-in-law and daughters-in-law.

Tanskanen and Danielsbacka investigate whether the investment of time and other resources received from parents is associated with the childbearing intentions of adult children. Using longitudinal data from Germany, Tanskanen and Danielsbacka find that in most cases parental investment does not increase children's intentions to have children. This indicates that in present-day Germany, parents may have a very limited influence on the fertility decisions of younger and middle-aged adults.

Dicks et al. examine family networks as a protective factor for young mothers neither in employment, education, or training (NEET) in the Netherlands. They show that having a partner and grandparents living in close proximity can prevent NEET status in young mothers or increase the likelihood of young mothers exiting a NEET status. They call for future studies to examine the effective amount of childcare provided and received and not just the availability of grandparents or formal childcare facilities.

Schnettler and Steinbach investigate how cross-household family complexity is associated with risk-taking behavior in adolescents. Using large-scale and cross-national data collected from 42 countries they include less frequently observed family structures (e.g., single father households). They also investigate several types of risk behavior in adolescents and find that post-separation family complexity is associated with higher incidences of risk-taking behavior in adolescents. The likelihood of risk-taking behavior is lowest in families with two biological parents and highest in two-household families where there are step-parents present in both households.

Arpino and Bellani focus on older women and their difficulties in managing both work and childcare responsibilities. They use longitudinal data from Europe and study the wellbeing of grandmothers who do and those who do not work. They find that non-working grandmothers benefit from childcare provision in terms of higher quality of life and a lower number of depressive symptoms, whereas working grandmothers do not experience any beneficial outcomes from caring for a grandchild. Older women may also experience difficulties when trying to combine work and family life.

Tammisalo et al. consider factors associated with the use of social media by older adults in Finland and compare their findings with those of their adult children. They find that several socio-demographic factors are associated with the use of social media. For instance, in both generations women used social media more likely than men and higher educated people more likely to use social media than people who are less educated. Social media use of adult children is also an important predictor for their parents' use of social media.

## Author Contributions

AT and MD wrote the article. AT drafted the article. Both authors contributed to the article and approved the submitted version.

## Funding

This study is part of NetResilience consortium funded by the Strategic Research Council at the Academy of Finland (grant number 345183) and INVEST flagship funded by the Academy of Finland (grant number 320162).

## Conflict of Interest

The authors declare that the research was conducted in the absence of any commercial or financial relationships that could be construed as a potential conflict of interest.

## Publisher's Note

All claims expressed in this article are solely those of the authors and do not necessarily represent those of their affiliated organizations, or those of the publisher, the editors and the reviewers. Any product that may be evaluated in this article, or claim that may be made by its manufacturer, is not guaranteed or endorsed by the publisher.
